# Rasch analysis of the hospital anxiety and depression scale (hads) for use in motor neurone disease

**DOI:** 10.1186/1477-7525-9-82

**Published:** 2011-09-29

**Authors:** Chris J Gibbons, Roger J Mills, Everard W Thornton, John Ealing, John D Mitchell, Pamela J Shaw, Kevin Talbot, Alan Tennant, Carolyn A Young

**Affiliations:** 1 Walton Centre for Neurology and Neurosurgery, Lower Lane, Liverpool, UK; 2 Department of Psychology, The University of Liverpool, Bedford Street South, Liverpool, UK; 3 Department of Neurology, Hope Hospital, Stott Lane, Greater Manchester, UK; 4 Royal Preston Hospital, Sharoe Green Lane, Preston, UK; 5 Sheffield Institute of Translational Neuroscience (SITraN), University of Sheffield, 385A Glossop Road Sheffield, UK; 6 Department of Clinical Neurology, John Radcliffe Hospital, Oxford, UK; 7 Academic Department of Rehabilitation Medicine, University of Leeds, Leeds General Infirmary, Leeds, UK

## Abstract

**Background:**

The Hospital Anxiety and Depression Scale (HADS) is commonly used to assess symptoms of anxiety and depression in motor neurone disease (MND). The measure has never been specifically validated for use within this population, despite questions raised about the scale's validity. This study seeks to analyse the construct validity of the HADS in MND by fitting its data to the Rasch model.

**Methods:**

The scale was administered to 298 patients with MND. Scale assessment included model fit, differential item functioning (DIF), unidimensionality, local dependency and category threshold analysis.

**Results:**

Rasch analyses were carried out on the HADS total score as well as depression and anxiety subscales (HADS-T, D and A respectively). After removing one item from both of the seven item scales, it was possible to produce modified HADS-A and HADS-D scales which fit the Rasch model. An 11-item higher-order HADS-T total scale was found to fit the Rasch model following the removal of one further item.

**Conclusion:**

Our results suggest that a modified HADS-A and HADS-D are unidimensional, free of DIF and have good fit to the Rasch model in this population. As such they are suitable for use in MND clinics or research. The use of the modified HADS-T as a higher-order measure of psychological distress was supported by our data. Revised cut-off points are given for the modified HADS-A and HADS-D subscales.

## Introduction

The Hospital Anxiety and Depression Scale (HADS) [1] is a reliable and potentially valid [2,3] measure for detecting depression and anxiety. The scale was designed to exclude measurement of somatic symptoms in medical outpatients; making it potentially suitable for use with motor neurone disease (MND) patients.

Due to the scale's apparent suitability, the HADS has been widely used in MND research for assessing states of anxiety and depression [4-7]. However, questions have been raised as to the suitability of the HADS depression subscale with MND patients as two previous studies [6,7] have omitted item D8 "I feel as though I am slowed down", on the reasonable assumption that responses to this item would be confounded by physical impairment. Whilst this change had clinical and face validity, in neither study was it accompanied by appropriate statistical or psychometric analysis to justify the alteration.

The Rasch model [8], a modern psychometric approach, ensures that the fundamental scaling properties of an instrument are assessed alongside traditional psychometric assessments of reliability and construct validity. The model operationalises the formal axioms of measurement [9] (order, unidimensionality and additivity), so allowing interval level data to be obtained from questionnaires. Rasch validation of the HADS has been proven useful in other clinical settings, such as rehabilitation [10] and Parkinson's disease [11].

The current study is a modern psychometric assessment of the HADS anxiety (HADS-A) and depression (HADS-D) subscales to assess the dimensionality, item suitability, reliability, scaling assumptions and internal consistency of the scales for use with MND patients. The analysis included evaluation of differential item functioning (DIF) by gender and age. In addition, the HADS total score (HADS-T) was investigated as a potentially valid measure of psychological distress in this population.

## Methods

### Main data collection

The psychometric and scaling properties of the HADS were assessed among 298 patients recruited from five regional MND care centres in the United Kingdom: The Walton Centre for Neurology and Neurosurgery in Liverpool, Preston Royal Hospital, Oxford John Radcliffe Hospital, Salford Hope Hospital, and Sheffield Royal Hallamshire Hospital. Participants all had a diagnosis of MND from a neurologist with expertise in MND. Patients were unselected for age, sex, and disease presentation or disability status. Questionnaires were either handed out during a routine clinic appointment or sent to the patients' home over a period of twelve months, along with a newsletter describing the research activities of their local care centre. Where patients were unable to complete the questionnaires by themselves a nurse or caregiver was allowed to act as a scribe. Informed consent was given by each participant.

Ethical permission was granted for this study from relevant hospital committees in the U.K. (Hammersmith 05/Q0401/7 and Tayside 07/S1402/64), and local research governance committees at all participating sites.

### Rasch Analysis

To evaluate the scaling properties and construct validity of the HADS, the Rasch measurement model was used [8]. Rasch analysis is a probabilistic mathematic modelling technique used to assess properties of outcome measures. Where data are shown to accord with model expectations, the internal construct validity of the scale is supported, and a transformation of ordinal data to interval scaling is possible [12].

For Rasch analysis, sample sizes requirements are influenced by scale targeting. For a scale that is well targeted (*i.e*. 40-60% endorsement rates for dichotomous items), a sample size of 108 will give accurate estimates of person and item locations (99% confidence of locations being within 0.5 logits). A sample size of 243 will provide accurate estimations of items and person locations irrespective of scale targeting [13].

Analyses used to assess whether the scale conformed to Rasch model expectations are briefly explained below. A comprehensive review with a more detailed explanation of the Rasch analytical process may be found elsewhere [10].

Rasch Unidimensional Measurement Model 2020 (RUMM2020) software (Version 4.1, Build 194) was used for the Rasch analyses presented in this study [14].

#### 1) Fit to the Rasch model

Rasch model fit is primarily indicated by a non-significant fit statistics, indicating that the scale does not deviate from model expectations. For example, both summary and individual item chi-square statistics should be non-significant, after adjusting for multiple testing. In addition, both person and item fit are assessed by their residual mean values. This examines the differences between the observed data and what is expected by the model for each person and each item estimate. At the summary level perfect fit is represented by a mean of zero and a SD of ± 1, while at the individual level for persons and items, a residual value between ± 2.5 is appropriate.

#### 2) Item difficulty and person ability

Estimates of a location on a common metric are provided for both persons (ability) and items (difficulty). In the context of the health sciences, 'ability' may be understood to represent the amount the person has of a given symptom, trait or feeling and difficulty may be understood to represent the magnitude of the symptom, trait or feeling represented by the item. For example, an item that reflected the sentiment that life was no longer worth living would be expected to represent a high level of depression when affirmed.

When data from a patient reported outcome scale is analysed through the Rasch model, both the items and persons are calibrated on the same metric that is measured in logits, or log-odds units. This allows for a comparison of the match between patients and items, showing whether or not the scale is well targeted. In the case of dichotomous items measuring depression, a patient with a logit value of zero on the depression scale would have a 50% chance of affirming an item whose level of depression (difficulty) was also at zero logits. A person with a level of depression at +2 logits (high depression) would have an 88% chance of affirming the item located at zero logits, whereas a person at -2 logits (low depression) would only have a 12% chance of affirming that item.

#### 3) Item category thresholds

The Rasch model allows for the analysis of the way in which response categories are understood by respondents. For example, in the case of a Likert style response as used in the HADS, some respondents may have difficulty differentiating between "Never" or "Very Rarely". In instances where there is too little discrimination between two response categories on an item, collapsing the categories into one response option can improve scale fit to the Rasch model.

Furthermore, where the same rating scale structure across items in not supported (*i.e*. where the distances between category thresholds vary across items) the unrestricted 'partial credit' Rasch polytomous model is used with conditional pair-wise parameter estimation [15].

#### 4) Local dependency

An assumption of the Rasch model is the local independence of items. A good example of this is where two stair climbing items are included in the same scale. If you can climb several flights of stairs unaided, you must be able to climb one flight of stairs. Such items are said to be locally dependent, and are not providing the same information as two independent items. This has the effect of spuriously inflating reliability, as well as affecting the parameter estimates of the Rasch model. This can be identified through the magnitude of residual item correlations, where items with residual correlations above 0.3 are considered to be locally dependent. The problem can be accommodated through the use of testlets, where the locally dependent items are simply added together into one 'super' item [16].

#### 5) Differential item functioning (DIF) [17]

Differential item functioning (DIF) occurs when different demographic or other contextual groups within the sample (*e.g*. males and females) respond in a different way to a certain question *when they have the same level of the underlying attribute*. Two types of DIF can be identified; uniform and non-uniform. Uniform DIF would occur, for example, when males respond consistently higher than females on an item, given the same level of depression. Non-uniform DIF would occur, for example, if females selected a higher response option to an item at lower levels of depression, compared to males, but a lower option at higher levels of depression. Differential item functioning is detected using analysis of variance (ANOVA, 5% alpha).

DIF was assessed for 3 contextual factors (called person factors within the Rasch analysis) including Location (Liverpool/Salford/Oxford/Sheffield/Preston), Age (Quartile split between participants, grouped < 55, 55-62,63-70, > 71) and Gender.

#### 6) Person separation index

The Person separation index (PSI) reflects the extent to which items can distinguish between distinct levels of functioning (where 0.7 is considered a minimal value for research use; 0.85 for clinical use) [18]. Where the distribution is normal, the PSI is equivalent to Cronbach's alpha.

#### 7) Unidimensionality

Finally, independent t-tests are employed to assess the final scale for unidimensionality. Two estimates are derived from subsets of items identified by a principal component analysis of the residuals, and the latent estimate of each person (and its standard error) calculated independently for each test. These estimates are then compared and the number of significant t-tests outside the ± 1.96 range indicates whether the scale is unidimensional or not. Generally, where less than 5% of the t-tests are significant this is indicative of a unidimensional scale (or the lower bound of the binomial confidence interval overlaps 5%) [19].

## Results

Summary demographic information and questionnaire response by centre is displayed in Table 1. This sample is broadly representative of the U.K. population of patients with MND [6,7].

**Table 1:** Demographics and Questionnaire Returns by Centre

Demographics	N = 298	n(%), M ± SD
	Age (years)	62.09 ± 11.01
	Sex: male	186 (62.4%)
	Questionnaires completed at home	278 (93.3%)
	Disease duration (years)	2.69 ± 3.54

Centre	Liverpool	110 (36.9%)
	Sheffield	38 (12.8%)
	Oxford	39 (13.1%)
	Salford	76 (25.5%)
	Preston	35 (11.7%)

### HADS-Depression

Initial fit to the Rasch model for the HADS-D subscale was poor (*χ*^2^(28) = 59.76 p < 0.01 - see Table 2 HADS-D Initial). Analysis of individual item fit statistics revealed that one item, item D8 "I feel as though I am slowed down" displayed a different level of fit to the other items. Whilst it appeared to have good fit statistics, all other items in the scale displayed classical misfit. Thus item D8 (Slowed down) was quantitatively different. This is an example of 'reverse fit indication' and the removal of item D8 (Slowed down) meant that the remaining 6 HADS-D items provided good fit to the Rasch model, *χ*^2^(24) = 39.90 p = 0.02 - see Table 2 HADS-D Final). Scale fit was marginally improved by collapsing disordered response categories for items D2 "I look forward with enjoyment to things" and D14 "I can enjoy a good book or radio or TV programme". Mild misfit was present for item D12 (Enjoyment) though this did not cause misfit to the Rasch model at the scale level and therefore the item was not removed. Analysis of variance tests revealed that all of the modified HADS-D scale items were free from DIF for location, age and sex.

**Table 2:** Summary Fit Statistics for Rasch Analyses

	# of items	Item Residual		Person Residual		Chi Square		PSI	Unidimensional t-test (CI %)	Extreme scores (%)
					
Analysis Name		Mean	± SD	Mean	± SD	Value	p			
HADS-D Initial	7	-0.19	1.51	-0.32	0.77	59.76	< 0.01	0.80	5.17% (2-7%)	0.30%
HADS-D Final	6	-0.15	1.30	-0.30	0.81	39.90	0.20	0.79	4.74% (2.4-8.1%)	15.11%
HADS-A Initial	7	0.14	2.09	-0.30	1.08	52.27	< 0.01	0.92	4.44% (2-7%)	1.70%
HADS-A Final	6	0.00	1.50	-0.36	1.01	34.75	0.07	0.84	5.07% (3-8%)	2.34%
HADS-T Initial	12	0.01	1.45	-0.21	0.90	113.92	< 0.01	0.86	9.90% (6-14%)	1.80%
HADS-T Final	10	-0.02	2.02	-0.47	0.87	10.51	0.23	0.76	7.37% (4-10%)	4.03%
***Ideal Values***		***0***	< 1.4*	***0***	***< 1.4***		***> 0.05^a^***	***> 0.85***	***< 5% (CI < 0.05)***	

Key: SD- Standard Deviation, p - Probability, PSI - Person Separation Index, CI - Confidence Interval

*= Can be inflated in the presence of testlets to accommodate local dependence.

**Figure 1:**
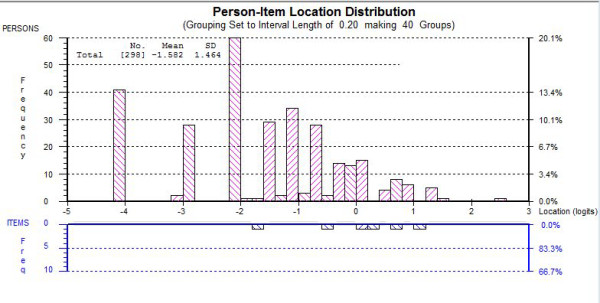
**Person-Item Threshold distribution for HADS-D subscale**.

The modified HADS-D exhibited a person separation index of 0.79, which is slightly lower than the suggested value of 0.85 for the scale to distinguish between distinct groups in a clinical setting [18]. But the fit statistic is likely to be affected by the skewed distribution shown in Figure 1.

Individual item statistics for the final HADS-D subscale, along with scoring structure, are given in Table 3.

**Table 3:** Item Fit Statistics and Scoring Structure for HADS-D

Item	Description	Location	SE	FitResid	ChiSq	Prob	Scoring structure
HADS-D2	Enjoy things	-1.36	0.08	-0.69	4.43	0.35	0-1-1-2
HADS-D4	Laugh	0.81	0.10	-1.26	4.63	0.33	0-1-2-3
HADS-D6	Cheerful	0.25	0.10	0.06	7.56	0.11	3-2-1-0
HADS-D10	Appearance	0.06	0.09	1.84	2.21	0.70	3-2-1-0
HADS-D12	Enjoyment	-0.40	0.09	-1.64	13.75	0.01	0-1-2-3
HADS-D14	Enjoy book	0.64	0.11	0.45	7.31	0.01	0-1-1-2

Key: SE - Standard Error, FitResid. = Fit Residual, ChiSq - Chi Square, Prob - Probability

### HADS-Anxiety

Initial fit of the anxiety scale to the Rasch model was poor (see Table 2 HADS-A Initial). Item A11"I feel restless as though I have to be on the move" displayed a high fit residual (4.18) and poor fit to the Rasch model (*χ*^2^(4) = 52.27 p < 0.01). Mild local dependency was shown between items A3 "I get a sort of frightened feeling as if something awful is about to happen" and A5 "Worrying thoughts go through my mind" (r = 0.33; p < 0.05). This local dependency was accommodated when the two items were collapsed into a testlet and, for this analysis, they would be considered as a single item. Removing item A11 improved fit to the Rasch model, with the six item solution (which included a pair of grouped items as above) providing acceptable fit statistics (see Table 2 HADS-A Final). Individual item fit statistics for the final HADS-A subscale are given in Table 4. All items in the HADS-A subscale were shown to be free from DIF for age, sex and location.

**Table 4:** Item Fit Statistics for HADS-A

Item	Description	Location	SE	FitResid	ChiSq	Prob
HADS-A1	Tense	-0.48	0.11	0.77	6.00	0.20
HADS-A3	Frightening	-0.30	0.09	-1.53	5.80	0.21
HADS-A5	Worrying	-0.60	0.09	-0.02	1.55	0.82
HADS-A7	Relaxed	0.08	0.10	2.13	8.10	0.09
HADS-A9	Butterflies	0.54	0.10	0.59	3.46	0.48
HADS-A13	Panic	0.76	0.11	-1.91	9.87	0.04

Key: SE - Standard Error, FitResid. = Fit Residual, ChiSq - Chi Square, Prob - Probability

Note: Scoring structure unchanged from original HADS-A

### HADS-Total

**Figure 2:**
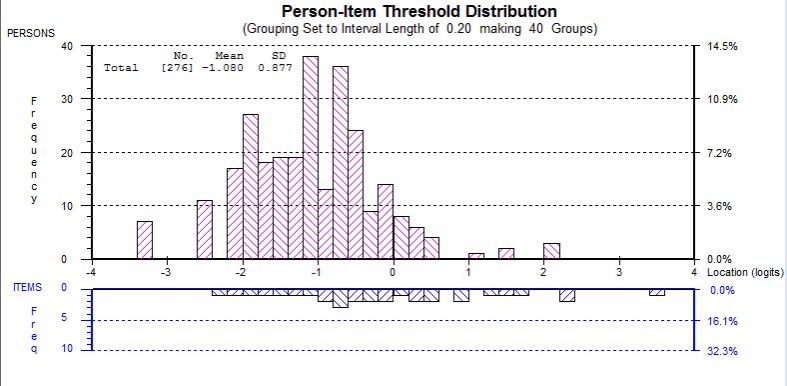
**Person item Distribution for HADS-T in MND**.

The viability of a HADS-T measure was explored by evaluating the scaling and psychometric properties of the 12 items remaining from the HADS-D and HADS-A subscales. Initial fit using the 12 items from the modified HADS-A and HADS-D subscales was unacceptable (*χ*^2^(48) = 113.92 p < 0.01, see Table 2 HADS-T Initial). Items D2 and D14 were rescored in the same manner as the HADS-D analysis. Following rescoring items D2 and D14, item D10 appeared to misfit the Rasch model. Removal of item D10 significantly improved fit to the Rasch model. Mild local dependency was present between items from the HADS-A and HADS-D subscales. Items from the HADS-A and HADS-D subscale were made into testlets to reduce the effect of the local dependency. Following this acceptable model fit was achieved (*χ*^2^(8) = 12.76, p = 0.12, see Table 2 HADS A Final). Unidimensionality was deemed acceptable with 7.37% (4-10%) of t-tests significant. Person separation index of 0.76 (α Cronbach's 0.78) was below the acceptable level for distinguishing between groups in a clinical context, and could not be explained by any floor effect as per the HADS-D analysis. Figure 2 shows that the HADS-T has a good spread of thresholds, indicating an excellent variation in item 'difficulty'. Item Fit statistics for the HADS-T are given in Table 5. Analysis of variance tests showed that the HADS-T measure of psychological distress was free from DIF by age, sex or location.

**Table 5:** Item Fit Statistics and Scoring Structure for HADS-T

Item	Description	Location	SE	FitResid	ChiSq	Prob	Scoring structure
HADS-A1	Tense	-0.69	0.10	0.44	3.76	0.44	3-2-1-0
HADS-D2	Enjoy things	-1.34	0.11	2.66	9.53	0.05	0-1-1-2
HADS-A3	Frightening	-0.50	0.09	1.45	3.40	0.49	3-2-1-0
HADS-D4	Laugh	1.14	0.11	-1.23	8.10	0.09	0-1-2-3
HADS-A5	Worrying	-0.77	0.09	-0.17	2.98	0.56	3-2-1-0
HADS-D6	Cheerful	0.52	0.10	0.65	8.13	0.09	3-2-1-0
HADS-A7	Relaxed	-0.14	0.10	-0.67	4.90	0.30	0-1-2-3
HADS-A9	Butterflies	0.21	0.10	1.12	2.28	0.38	0-1-2-3
HADS-D12	Enjoyment	-0.29	0.09	3.11	11.34	0.02	0-1-2-3
HADS-A13	Panic	0.45	0.1	-2.03	9.91	0.05	3-2-1-0
HADS-D14	Enjoy good	1.42	0.15	-0.27	2.32	0.68	0-1-1-2

Key: SE - Standard Error, FitResid. = Fit Residual, ChiSq - Chi Square, Prob - Probability

#### Modified cut-off points

**Figure 3:**
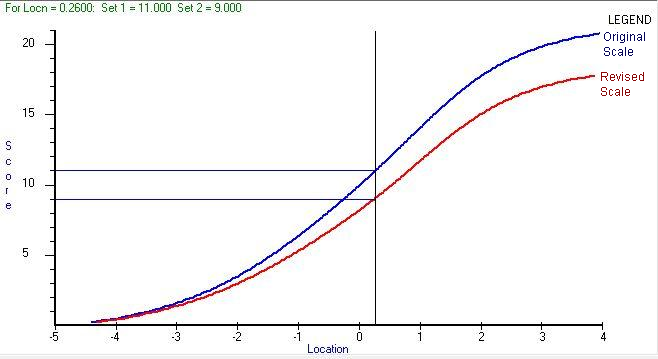
**Example of equating tests to ascertain new cut-off scores for the HADS-A**.

Rasch analysis allows for the transformation of scores between the raw questionnaire scores and post-Rasch estimates. Table 6 shows the relationship between cut-off points suggested by Zigmond and Snaith [1] on their original scale and on the revised scale. Cut off points were originally suggested as 11 or greater for case levels of depression or anxiety, 8-10 for borderline cases and scores of 7 or lower representing non-cases. An example of the process whereby new cut-off points are ascertained for the HADS-A is given in Figure 3. A scale score of 11 (probable anxiety) on the original scale equates to a person location of 0.26 logits on the latent estimate of anxiety. Equating this person location to the revised scale gives a new cut-off point of 9.

**Table 6:** Equated Cut-off points

	Original cut-off	N	%	Revised cut-off	N	%
HADS-D	≥ 11	45	15.1	≥ 8	33	11.1
	8 to 10	54	18.1	5 to 7	61	20.5
	≤ 7	199	66.8	≤ 4	204	68.5

HADS-A	≥ 11	56	18.8	≥ 9	67	22.5
	8 to 10	52	17.5	7 to 8	33	11.1
	≤ 7	190	63.8	≤ 6	198	66.4

For the HADS-T total score of mood disorder, equating tests suggest cut-off points of 17 for 'possible' mood disorder and 21 for 'probable' mood disorder (10).

Table 6 shows that the original HADS-D was slightly overestimating the prevalence of probable (together) depression, which could be driven by the inclusion of the item D8 (slowed down) in the original scale to which 88% of respondents scored highly. Likewise equating scores for the HADS-A subscale revealed that possible and probable levels of anxiety were being overestimated by the original scale due to the inclusion of item A11 "I feel restless as if I have to be on the move". The revised scale also increases the level of 'probable' anxiety while the 'possible' levels fall sharply.

## Discussion

The HADS is commonly used in MND clinics and has been used in a number of past studies in MND [4-7]. Rasch analysis of the scale makes an important contribution to current understanding of the measurement properties of the HADS in MND.

The results of our study indicate that the standard 7-item measure of depression should be modified for use in MND due to the confounding effect of item D8 "I feel slowed down" causing overestimation of possible cases of depression in this population. Following the removal of item D8 the HADS-D subscale showed good fit to the Rasch model, including acceptable dimensionality. The removal of this item mirrors alterations made to the HADS-D subscale by other researchers working in MND [6,7] who felt the item confounded with the high levels of impairment frequently witnessed in the disease. Reliability for the depression subscale in the current study was below the recommended threshold for clinical use [18], and may have been affected by the large floor effect in our sample. A floor effect may be expected when administering a depression scale to a population that have been shown to have a low incidence of depression [20]. Analysis of the person-item distribution for the depression subscale reveals scale information to be maximised around the clinical cut off points, indicating that the floor effect does not impact upon the usefulness of the modified HADS.

In the current study problems were also evident in the original HADS-A, where one item "I feel restless as if I have to be on the move" was found to misfit the Rasch model. The removal of this item yielded a reliable 6-item solution satisfying Rasch model expectations, including the assumption of unidimensionality. The level of reliability of this modified scale makes it suitable for estimation of anxiety states both in clinic or when used in research. It was also shown to be free from item bias by gender, age or location. With the revised cut points for the modified HADS-A, it was shown that the original anxiety subscale overestimate anxiety states.

Previous research has identified that the HADS-D and HADS-A subscales are highly correlated and have suggested that a HADS-T measure could be a single higher order factor corresponding to psychological distress or negative affectivity [3,21]. This has been statistically supported by some studies [10,22-24]. Likewise the current study gave support to the use of a modified HADS-T total score as a measure of psychological distress in this population. The low person separation index demonstrated by this scale suggests that it is suitable as a summary scale for research, rather than for clinical use. This may have been as a result of the narrower operational range of the scale caused by removing the effects of local dependency in the data through the testlet design.

The performance of the HADS has been called into question following Rasch analysis in other conditions. In patients with cancer, the HADS requires some modification (removal of items D5, D7 and A6) in order to satisfy the demands of the Rasch model [25]. Likewise, the anxiety item A6 "I feel restless as if I have to be on the move" was also found to misfit the Rasch model when the HADS was tested in a population of 296 musculoskeletal outpatients [10]. In Parkinson's disease the original depression subscale was deemed unsuitable for use and could not be successfully modified to fit the Rasch model [11]. Conversely, in a Chinese sample of stroke patients, the depression subscale of the HADS displayed adequate fit to the Rasch model [26]. The variability of the results of Rasch analyses across a range of diseases suggests that the performance of the HADS may vary by diagnostic group and reinforces the need for clinicians and researchers to formally test the psychometric properties of the instruments they intend to use on different diagnostic groups [27].

For clinical guidance, revised cut-off values are required to indicate clinical case status for depression and anxiety for the new Rasch validated modified scales that have been suggested. Such values are provided for the original scales. We provide these for the new scales by simple mathematical equivalence, accounting for the reduced number of items. While revised values have not been subject to validation by clinical diagnostic interview, the suggested prevalence of case-level depression in our sample (11.1%) is similar to the pooled prevalence estimate of 9.7% (range 9-11) taken from three studies in MND that used DSM-IV criteria for diagnosis of current major depressive episode (MDE) [28-30]. The current study may have been improved by validating the revised cut-off values by clinical diagnostic interview.

These findings support the use of the modified HADS-D and HADS-A for use with patients with MND within clinics and research, and support the modified HADS-T for research use where necessary. All three measures displayed internal construct validity and had no gender or age related item bias.

## Conflicts of Interest

The authors declare that they have no competing interests.

## Authors' contributions

CJG collected data, conducted analyses and is the primary author of this paper.

EWT assisted in study design and authoring of the paper. Co-grant holder.

RJM provided expert review and assisted in study design and editing.

JE, JDM, PJS and KT facilitated data collection in the MND care centres they run.

AT provided expert statistical advice regarding Rasch analysis.

CAY assisted in study design, authoring, collection of data and editing. Primary grant holder.

All authors read and approved the final version of this manuscript.
